# Genetic analysis reveals diversity and genetic relationship among *Trichoderma* isolates from potting media, cultivated soil and uncultivated soil

**DOI:** 10.1186/s12866-015-0483-8

**Published:** 2015-07-28

**Authors:** Abdullah M. Al-Sadi, Fatma A. Al-Oweisi, Simon G. Edwards, Hamed Al-Nadabi, Ahmed M. Al-Fahdi

**Affiliations:** Department of Crop Sciences, College of Agricultural and Marine Sciences, Sultan Qaboos University, Muscat, Oman; Royal Court Affairs, Seeb, Oman; Department of Crop and Environment Sciences, Harper Adams University, Newport, , TF10 8NB, , UK; The Botanic Garden, College of Science, Sultan Qaboos University, Muscat, Oman

**Keywords:** Biological control, Fallow soil, Phylogenetic analysis

## Abstract

**Background:**

*Trichoderma* is one of the most common fungi in soil. However, little information is available concerning the diversity of *Trichoderma* in soil with no previous history of cultivation. This study was conducted to investigate the most common species and the level of genetic relatedness of *Trichoderma* species from uncultivated soil in relation to cultivated soil and potting media.

**Results:**

A total of 24, 15 and 13 *Trichoderma* isolates were recovered from 84 potting media samples, 45 cultivated soil samples and 65 uncultivated soil samples, respectively. Analysis based on the internal transcribed spacer region of the ribosomal RNA (rRNA) and the translation elongation factor gene (EF1) indicated the presence of 9 *Trichoderma* species: *T. harzianum* (16 isolates)*, T. asperellum* (13)*, T. citrinoviride* (9)*, T. orientalis* (3), *T. ghanense* (3)*, T. hamatum* (3)*, T. longibrachiatum* (2), *T. atroviride* (2)*,* and *T. viride* (1)*.* All species were found to occur in potting media samples, while five *Trichoderma* species were recovered from the cultivated soils and four from the uncultivated soils. AFLP analysis of the 52 *Trichoderma* isolates produced 52 genotypes and 993 polymorphic loci. Low to moderate levels of genetic diversity were found within populations of *Trichoderma* species (H = 0.0780 to 0.2208). Analysis of Molecular Variance indicated the presence of very low levels of genetic differentiation (Fst = 0.0002 to 0.0139) among populations of the same *Trichoderma* species obtained from the potting media, cultivated soil and uncultivated soil.

**Conclusion:**

The study provides evidence for occurrence of *Trichoderma* isolates in soil with no previous history of cultivation. The lack of genetic differentiation among *Trichoderma* populations from potting media, cultivated soil and uncultivated soil suggests that some factors could have been responsible for moving *Trichoderma* propagules among the three substrates. The study reports for the first time the presence of 4 *Trichoderma* species in Oman: *T. asperellum, T. ghanense, T. longibrachiatum* and *T. orientalis.*

## Background

*Trichoderma* species are free living fungi that are commonly found in soil and woody materials. They are symbionts on plants and play an important role as antagonistic fungi towards pathogenic fungi [1, 2]. *Trichoderma* species are generally characterized by their rapid growth and ability to survive in variable environmental conditions. They are commonly found in agricultural lands, forests, and deserts. They are important contributors in the decomposition of plant materials. Some species of *Trichoderma* are economically important because of their ability to produce industrial enzymes and antibiotics [3, 4].

Soil-based system is the preferable choice for production of vegetables in the Arabian Peninsula and in different parts of the world. However, due to poor soil characteristics [5–7], many growers tend to use potting media and organic fertilizers in the production system of vegetables. In addition, they also replace farm soil with uncultivated soil (fallow soil) in order to reduce populations of soilborn fungal pathogens [7]. *Trichoderma* species are known to be very common in cultivated soils and potting media [8–11]. However, no information is available concerning diversity of *Trichoderma* in uncultivated soil and their relationship to *Trichoderma* species from cultivated soil and potting media. This makes it difficult for growers to know the impact of soil replacement on lowering *Trichoderma* diversity in farm soil.

Populations of fungi can vary in their levels of genetic diversity from one species to the other [12], one geographical region to the other [13] and between different cultivation systems [14]. As a result, several molecular markers have been developed and used to characterize the level of genetic diversity within and among fungal populations. These include the use of isozyme variation, Restriction Fragment Length Polymorphism (RFLP), Amplified Fragment Length Polymorphism (AFLP) and Random Amplified Polymorphic DNA (RAPD). AFLP has been found to be powerful in the characterization of genetic diversity among and within populations of different fungi [15, 16].

This study was conducted to characterize the genetic diversity of *Trichoderma* species in uncultivated soils and their genetic relationship with *Trichoderma* species isolated from cultivated soils and potting media. Results will provide a basis for future studies on *Trichoderma* in soil and potting media.

## Results

### Analysis of *Trichoderma* species in soil and potting media samples

**Fig. 1:**
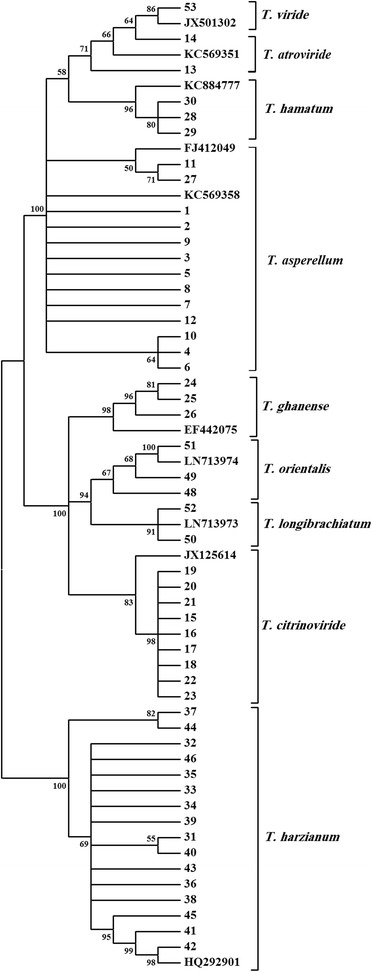
Phylogram representing the relationship of 52 *Trichoderma* isolates to sequences of 9 reference isolates. The analysis is based on the ITS rDNA sequences inferred by a neighbor-joining method search. Numbers within the tree represent the bootstrap values (values above 50 % are indicated; 1000 replications)

**Fig. 2:**
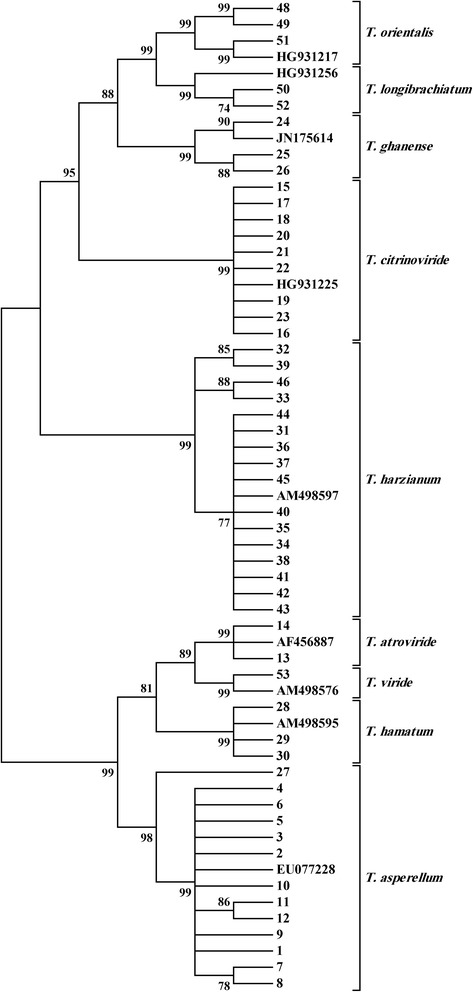
Phylogram representing the relationship of 52 *Trichoderma* isolates to sequences of nine reference isolates. The analysis is based EF sequences inferred by a neighbor-joining method search. Numbers within the tree represent the bootstrap values (values above 50 % are indicated; 1000 replications)

**Fig. 3:**
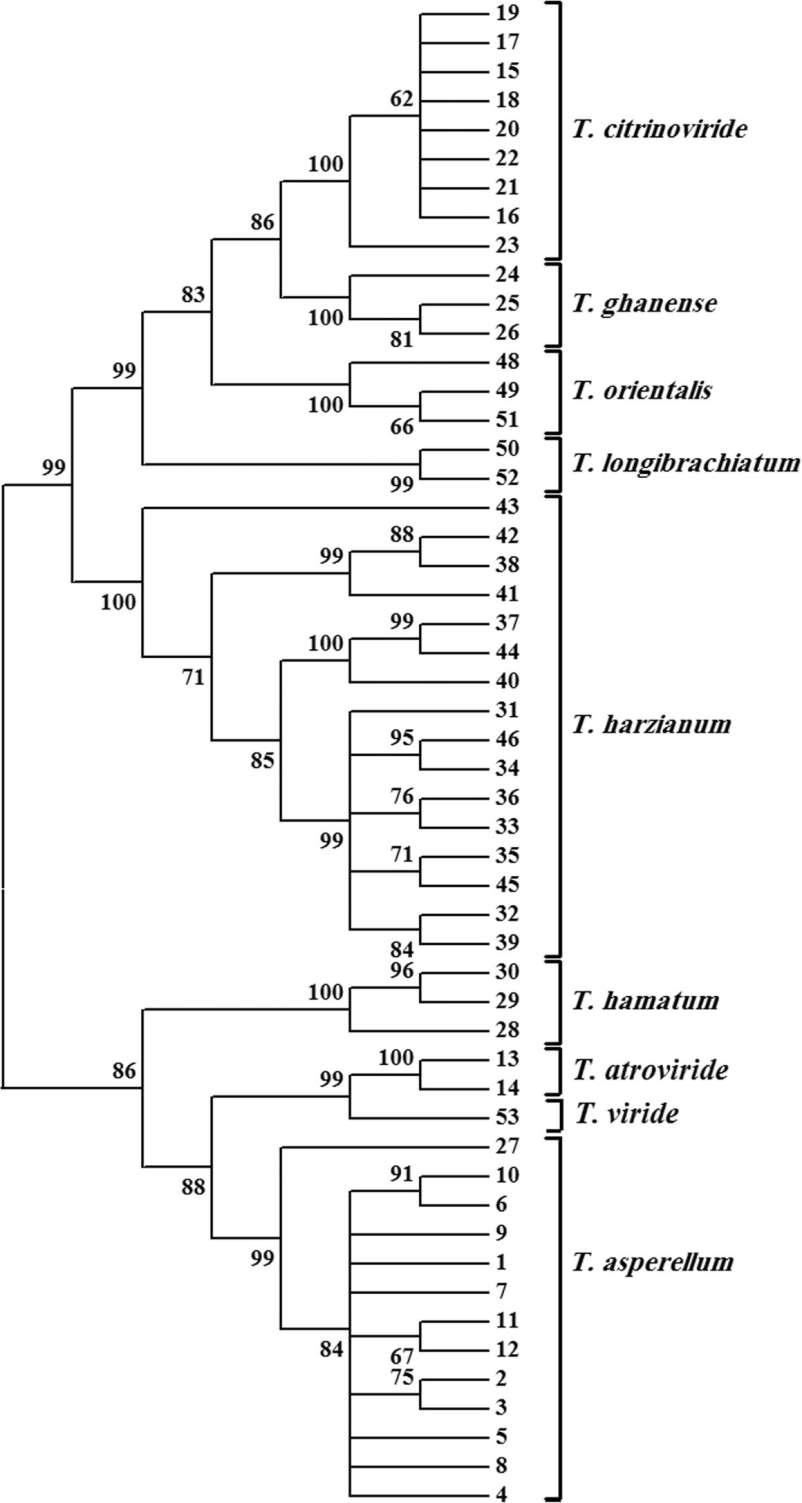
Phylogram representing clustering of 52 *Trichoderma* isolates based on combined analysis using ITS rDNA and EF sequences inferred by a neighbor-joining method search. Numbers within the tree represent the bootstrap values (values above 50 % are indicated; 1000 replications)

Phylogenetic analysis based on the ITS rDNA and EF1 sequences separated *Trichoderma* isolates into clusters representing 9 species: *T. asperellum* (13 isolates)*, T. atroviride* (2)*, T. citrinoviride* (9)*, T. ghanense* (3)*, T. hamatum* (3)*, T. harzianum* (16)*, T. longibrachiatum* (2), *T. orientalis* (3) and *T. viride* (1) (Figs. 1, 2, 3)*.*

All species were found to occur in potting media samples (24 isolates obtained from 84 samples), with *T. citrinoviride* being the most common (8), followed by *T. hamatum* (3), *T. harzianum* (3), *T. asperellum* (3), *T. atroviride* (2), *T. ghanense* (2), *T. longibrachiatum* (1), *T. orientalis* (1), and *T. viride* (1).

Isolations from cultivated soil (15 isolates obtained from 45 samples) revealed the presence of *T. asperellum* (7)*, T. harzianum* (5)*, T. citrinoviride* (1)*, T. ghanense* (1)*,* and *T. orientalis* (1). On the other hand, isolations from the uncultivated soils (13 isolates obtained from 65 samples) showed that the most common species were *T. harzianum* (8), *T. asperellum* (3), *T. orientalis* (1) and *T. longibrachiatum* (1).

The ITS rRNA gene showed higher level of intraspecific variation compared to the EF1 gene, which gave higher resolution in separating *Trichoderma* species. The bootstrap support for separating species from each other was 99 % for the EF1 gene (Fig. 2). However, the bootstrap support for separating species based on the ITS rRNA gene showed that it ranges from 67 to 100 %, with one species (*T. asperellum*) not forming a tight cluster (Fig. 1). When the sequences of ITS rDNA and EF1 were used to produce a single tree, the resolution in separating *Trichoderma* species imporved to 99–100 % bootstrap support (Fig. 3).

No relationship was observed between clustering of the isolates based on ITS rDNA or EF1 sequences and clustering based on the substrates from which the isolates were obtained (Figs. 1, 2; Table 1). For example, *T. harzianum* isolates grouped into several sub-clusters. However, neither ITS-based sub-clustering, nor EF1-based sub-clustering correlated with the substrates from which the isolates were obtained. All ITS and EF1 sequences were deposited in the European Nucleotide Archive (Table 1).

**Table 1:** Characteristics of *Trichoderma* species collected from cultivated and uncultivated soils and potting media

Isolate No.	Identity	Accession # (ITS rRNA)^a^	Accession # (EF-1)^a^	Substrate	Cultivated species^b^	Country^b^
1	*T. asperellum*	LN846676	LN846728	Uncultivated soil	-	Oman
2	*T. asperellum*	LN846677	LN846729	Cultivated soil	*Cucumis sativus*	Oman
3	*T. asperellum*	LN846678	LN846730	Cultivated soil	*Cucumis sativus*	Oman
4	*T. asperellum*	LN846679	LN846731	Cultivated soil	*Cucumis sativus*	Oman
5	*T. asperellum*	LN846680	LN846732	Cultivated soil	*Cucumis sativus*	Oman
6	*T. asperellum*	LN846681	LN846733	Cultivated soil	*Phoenix dactylifera*	Oman
7	*T. asperellum*	LN846682	LN846734	Uncultivated soil	-	Oman
8	*T. asperellum*	LN846683	LN846735	Cultivated soil	*Phoenix dactylifera*	Oman
9	*T. asperellum*	LN846684	LN846736	Potting media	-	Latvia
10	*T. asperellum*	LN846685	LN846737	Cultivated soil	*Lycopersicum esculentum*	Oman
11	*T. asperellum*	LN846686	LN846738	Uncultivated soil	-	Oman
12	*T. asperellum*	LN846687	LN846739	Potting media	-	Latvia
13	*T. atroviride*	LN846688	LN846740	Potting media	Estonia	Estonia
14	*T. atroviride*	LN846689	LN846741	Potting media		Germany
15	*T. citrinoviride*	LN846690	LN846742	Cultivated soil	*Cucumis sativus*	Oman
16	*T. citrinoviride*	LN846691	LN846743	Potting media	-	Oman
17	*T. citrinoviride*	LN846692	LN846744	Potting media	-	Oman
18	*T. citrinoviride*	LN846693	LN846745	Potting media	-	Estonia
19	*T. citrinoviride*	LN846694	LN846746	Potting media	-	Estonia
20	*T. citrinoviride*	LN846695	LN846747	Potting media	-	Estonia
21	*T. citrinoviride*	LN846696	LN846748	Potting media	-	Finland
22	*T. citrinoviride*	LN846697	LN846749	Potting media	-	The Netherlands
23	*T. citrinoviride*	LN846698	LN846750	Potting media	-	UK
24	*T. ghanense*	LN846699	LN846751	Potting media	-	Estonia
25	*T. ghanense*	LN846700	LN846752	Potting media	-	Estonia
26	*T. ghanense*	LN846701	LN846753	Cultivated soil	*Phoenix dactylifera*	Oman
27	*T. asperellum*	LN846702	LN846754	Potting media	Estonia	Estonia
28	*T. hamatum*	LN846703	LN846755	Potting media	-	The Netherlands
29	*T. hamatum*	LN846704	LN846756	Potting media	-	Estonia
30	*T. hamatum*	LN846705	LN846757	Potting media	-	Estonia
31	*T. harzianum*	LN846706	LN846758	Potting media	-	Finland
32	*T. harzianum*	LN846707	LN846759	Potting media	-	The Netherlands
33	*T. harzianum*	LN846708	LN846760	Cultivated soil	*Cucumis sativus*	Oman
34	*T. harzianum*	LN846709	LN846761	Uncultivated soil	-	Oman
35	*T. harzianum*	LN846710	LN846762	Cultivated soil	*Medicago sativa*	Oman
36	*T. harzianum*	LN846711	LN846763	Cultivated soil	*Phaseolus vulgaris*	Oman
37	*T. harzianum*	LN846712	LN846764	Uncultivated soil	Dam soil	Oman
38	*T. harzianum*	LN846713	LN846765	Cultivated soil	*Solanum tuberosum*	Oman
39	*T. harzianum*	LN846714	LN846766	Uncultivated soil	Dam soil	Oman
40	*T. harzianum*	LN846715	LN846767	Uncultivated soil	Dam soil	Oman
41	*T. harzianum*	LN846716	LN846768	Uncultivated soil	Dam soil	Oman
42	*T. harzianum*	LN846717	LN846769	Cultivated soil	*Phoenix dactylifera*	Oman
43	*T. harzianum*	LN846718	LN846770	Uncultivated soil	Dam soil	Oman
44	*T. harzianum*	LN846719	LN846771	Uncultivated soil	Dam soil	Oman
45	*T. harzianum*	LN846720	LN846772	Potting media	-	Oman
46	*T. harzianum*	LN846721	LN846773	Uncultivated soil	-	Oman
48	*T. orientalis*	LN846722	LN846774	Potting media	-	Oman
49	*T. orientalis*	LN846723	LN846775	Uncultivated soil	-	
50	*T. longibrachiatum*	LN846724	LN846776	Uncultivated soil	Dam soil	Oman
51	*T. orientalis*	LN846725	LN846777	Cultivated soil	*Capsicum annuum*	Oman
52	*T. longibrachiatum*	LN846726	LN846778	Potting media	-	Germany
53	*T. viride*	LN846727	LN846779	Potting media	-	Germany

^**a**^Accession number of sequences deposited in the European Nucleotide Archive

^**b**^The sign (−) indicates that the soil was not cultivated with any plant species/no vegetation

### Analysis of diversity within populations of *Trichoderma* species

Analysis of 52 *Trichoderma* isolates using 3 primer-pair combinations produced 993 polymorphic loci (100 % polymorphism), with the percentage of polymorphic loci ranging from 16 % to 80 % for the different *Trichoderma* species. The number of polymorphic loci produced by the three primer combinations were 277 (*Eco*RI-AGA/*Mse*I-CAT), 389 (*Eco*RI-AGT/*Mse*I-CAT) and 327 (*Eco*RI-AGT/*Mse*I-CAA). The three primer pair combinations also resulted in moderate levels of Nei’s gene diversity, which were 0.1732, 0.2632 and 0.1810 respectively.

**Fig. 4:**
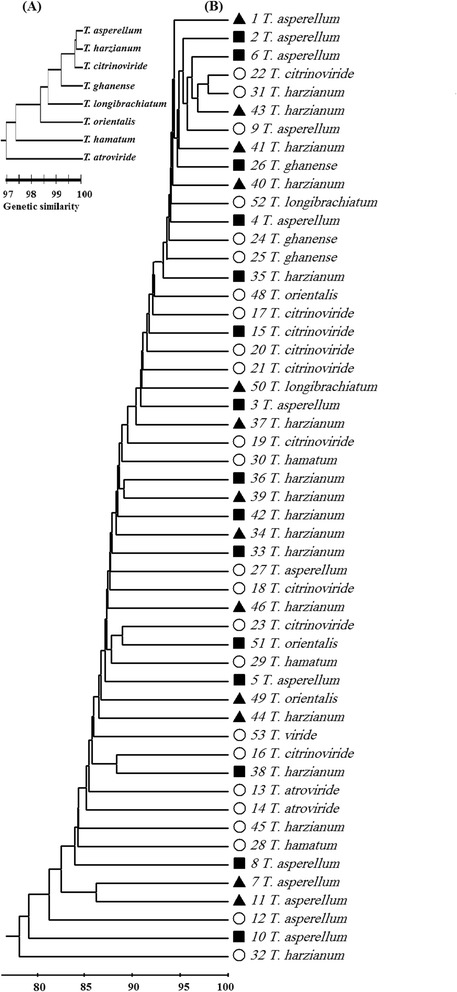
UPGMA dendrogram illustrating genetic similarity [31] among isolates and species of *Trichoderma* from potting media, cultivated soils and uncultivated soils based on AFLP fingerprinting analysis: **a** shows grouping based on species while **b** shows grouping based on isolates. The signs indicate that the isolates originate from cultivated soil (square sign), uncultivated soil (triangle sign) or potting media (circle sign)

AFLP analysis of the 52 *Trichoderma* isolates produced 52 different AFLP genotypes (Table 2; Fig. 4). Each isolate representing a genotype differed from the others by at least 155 alleles. *Trichoderma* isolates showed a moderate level of genetic diversity (H = 0.2110). *Trichoderma viride* was excluded from population specific analysis of genotypic and genetic diversity because it consisted of one isolate. The seven populations differed in their level of gene diversity. The percent polymorphic loci ranged from 16 to 80 % and Nei’s gene diversity estimates ranged from 0.0780 to 0.2208 (Table 2).

**Table 2:** Genotypic and genetic analysis of *Trichoderma* species obtained from potting media, uncultivated soils and cultivated soils in Oman

Species	N	G	NPL	PPL	H
All	52	52	993	100	0.2110
*T. asperellum*	13	13	799	80	0.2208
*T. atroviride*	2	2	225	23	0.1133
*T. citrinoviride*	9	9	571	58	0.1682
*T. ghanense*	3	3	260	26	0.1164
*T. hamatum*	3	3	363	37	0.1625
*T. harzianum*	16	16	767	77	0.2036
*T. orientalis*	3	3	323	33	0.1446
*T. longibrachiatum*	2	2	155	16	0.0780

*N* numbers of individuals, *G* genotypes, *NPL* numbers of polymorphic loci, *PPL* percentage of polymorphic loci (out of 993), *H* the gene diversity by Nei [30]

### Genetic similarity and cluster analysis

The level of genetic similarity among the seven populations was found to vary from 92 to 99 %. *T. asperellum, T. harzianum* and *T. citrinoviride* were found to share a high level of genetic similarity (Fig. 4a). *Trichoderma atroviride* shared the least level of genetic similarity with other *Trichoderma* species. Cluster analysis showed that *T. asperellum, T. harzianum*, *T. citrinoviride*, *T. ghanense* and *T. longibrachiatum* clustered together (Fig. 4a). Cluster analysis of the three most common species (*T. asperellum, T. harzianum*, and *T. citrinoviride*) showed that there was no relationship between clustering of isolate of the same species and the substrata from which they were isolated (Fig. 4b). In addition, there was no relationship between clustering of *Trichoderma* isolates and the species they belong to.

### Partition of genetic variation

Analysis showed that the percent genetic variation is 1.39 % among populations of *T. asperellum* obtained from potting media, cultivated soil and uncultivated soil (F = 0.0139; *P* = 0.3059). The percent genetic variation among populations of *T. harzianum* obtained from potting media, cultivated soil and uncultivated soil was found to be 0.02 % (F = 0.0002; *P* = 0.4575). These values indicate that most of the genetic variation is within populations of the same species. They also indicate that the level of gene flow among populations of fungal isolates is high among potting media, cultivated soil and uncultivated soil.

## Discussion

*Trichoderma* is a widespread genus of fungi. Previous studies in Oman provided evidence for occurrence of *T. hamatum* in the greenhouse soil [5] and *T. harzianum,* and *T. parceramosum* in desert crusts [17]. Our current investigation revealed presence of 9 *Trichoderma* species in potting media samples, 5 species in cultivated soil samples and 4 species in uncultivated soil samples. The higher level of diversity in *Trichoderma* species in potting media is related to the fact that these material consist of different organic and inorganic ingredients including peat moss, sphagnum, shredded bark, sawdust, vermiculite, perlite, clay and sand [18]. The *Trichoderma* species could have therefore been naturally occurring in these products or they could have been introduced after composting [18, 19].

Identification of *Trichoderma* to the species level based on reference sequences from the National Center for Biotechnology Information correlated with phylogenic analysis based on sequences of the ITS rRNA and EF1 genes. However, the limited intraspecific variation within *Trichoderma* species based on sequences of the EF1 gene helped gave better resolution in separating *Trichoderma* species when compared to sequences of the ITS region. The EF1 gene, especially when combined with ITS rDNA data, can give better resolution and is increasingly used in the identification of several fungal species [16, 20].

The frequency of isolation of *Trichoderma* from cultivated soil (33 %) was higher than that from uncultivated soil (20 %). Since none of the sampled farms add commercial products of *Trichoderma* to soil, *Trichoderma* isolates could have been naturally occurring in the cultivated soil. Another possible source of *Trichoderma* into farms is the use of potting media. Many farmers in Oman use potting media products for germination of vegetable crops or in soil [7, 11]. Since findings from this study and from previous studies indicated that potting media act as a source of *Trichoderma* [10], it is possible that potting media contributed to moving *Trichoderma* isolates to cultivated soils. This is supported by AMOVA analysis which indicated the presence of low levels of genetic differentiation and high levels of gene flow among *Trichoderma* populations obtained from cultivated soil and potting media (Fst = 0. 01389 for *T. asperellum* and 0.00016 for *T. harzianum*).

Our study showed the occurrence of four *Trichoderma* species in soils with no previous history of cultivation. Wind driven sand, which is common is Oman, could have contributed to moving some *Trichoderma* propagules to areas without vegetation [7]. The lack of relationship between AFLP based clustering of *Trichoderma* isolates and their origin as well as the insignificantly (P > 0.05) very low levels of genetic differentiation among populations of the same species obtained from cultivated and uncultivated soils may explain the hypothesis of movement of *Trichoderma* isolates between cultivated and uncultivated soils. In addition, some of the *Trichoderma* isolates were recovered from soil trapped behind dams. During rainy periods in Oman, which are very limited in this part of the world, the flowing water from mountainous areas is usually trapped behind dams. Since it is common to find *Acacia* spp. and some other plants in the way of the flowing water, it is possible that *Trichoderma* propagules could have been moved by the flowing water and then deposited in the dam soil. A study is in progress to characterize naturally occurring fungal species in the path of flowing water and from the rhizosphere of wild plants.

Farmers in Oman usually replace farm soils with uncultivated soils imported from soils trapped behind dams or non-cultivated lands. Besides reducing pathogen inoculum in farms [7], this practice could help introduce beneficial *Trichoderma* isolates, especially *T. asperellum* and *T. harzianum,* into farms.

Several species of *Trichoderma* have been long used as biological control agents to manage diseases of vegetable and other crops [10, 21]. *Trichoderma harzianum* and *T. asperellum* are the two most common species and were isolated from potting media, cultivated soil and uncultivated soil. *T. harzianum* is a common species in soil and potting media and it is commercially used as a biological control agent [10, 21]. *T. asperellum* has been used for induction of resistance to different diseases [22, 23]. The relatively high level of occurrence of *T. asperellum* and *T. harzianum* in Oman soils (82 % of the total isolates) might contribute to improving soil health through disease suppression in different crop systems

The overall level of genetic diversity of *Trichoderma* isolates was found to be moderate (H = 0.2110). The level of genetic diversity for the different species varied from 0.1133 to 0.2208. However, clustering based on AFLP data did not correlate with sources of the isolates, nor with clustering based on the ITS rRNA and the EF1 genes. Variation in the level of genetic diversity and sequences among the different species could be related to several reasons. Multiple introductions of *Trichoderma* isolates into Oman could have played a major role in augmenting the genetic diversity of *Trichoderma* populations [15, 16, 24]. The hypothesis of multiple introductions is supported by the formation of several AFLP genotypes and the lack of relationship between AFLP data and the source of the isolates. It is also supported by the lack of relationship between ITS/EF1 based clustering and the sources of *Trichoderma* isolates. This may indicate that the isolates were introduced at different times and from different sources. Other factors which could have affected the level of genetic diversity is the differences in the reproduction modes and the level of sexual recombination between the different species [15, 16].

## Conclusion

This study provided evidence that the diversity in *Trichoderma* species varies from one substrate to the other. This is the first report showing *T. asperellum, T. ghanense, T. longibrachiatum* and *T. orientalis* in Oman*.* AFLP analysis suggested possible introductions of *Trichoderma* isolates from potting media into cultivated soil and also movement of *Trichoderma* between cultivated and uncultivated soils. The relatively high level of diversity in *Trichoderma* species and isolates may give an indication about the health of soil and its ability to contribute in the suppression of soilborn diseases that affect different crops. In addition, the high diversity may make it possible to find and select *Trichoderma* isolates with high antagonistic properties. Studies are in progress to characterize the biological properties of some of the *Trichoderma* isolates obtained in this study. Future studies are required to focus on characterizing *Trichoderma* isolates and other biocontrol agents from extreme environments in order to come up with isolates that can tolerate the harsh conditions of arid countries.

## Methods

### Collection of *Trichoderma*

*Trichoderma* isolates were obtained from potting media, cultivated soil and uncultivated soil. Twenty four *Trichoderma* isolates were obtained from 84 potting media products originating from Oman, The Netherlands, Estonia, Germany, Finland, Latvia, or UK (trade names are kept anonymous). Isolations from these products were done using direct plating technique as previously described by Al-Sadi et al. [11].

Fifteen *Trichoderma* isolates were obtained from 45 samples of cultivated (farm) soils planted with date palm, cucumber, tomato, bean, alfalfa, pepper or potato. This was done by collecting approximately 50 g soil sample from the top 15 cm of the rhizosphere of each plant species. The samples were collected from Barka, to the North-West of Muscat and from Seeb during 2013.

*Trichoderma* isolates (13) were also obtained from 65 uncultivated soil samples with no known history of previous cultivation. Collection of soil samples from uncultivated soil was done by collecting 50 g soil from the top 15 cm of soil (Table 1). The sites from which the samples were collected were 5–17 Km away from the coastal area of Barka and Seeb districts. The sites have no previous history of cultivation with any crop and the temperature ranges from 15 °C in the winter to 49 °C in summer (avg. 32 °C). Collection of soil samples from cultivated and un-cultivated soil was during the period from April to November 2013.

### Isolation and identification of *Trichoderma*

Isolation of *Trichoderma* from soil samples was achieved using direct plating as described by Al-Sadi et al. [11]. Approximately 100–150 mg of soil sample was spread on the surface of 2.5 % potato dextrose agar (PDA) amended with 50 mg l^−1^ Rose Bengal. Each soil was plated onto three replicate Petri dishes and the Petri dishes were incubated at 25 °C for 5–7 d. Growth of fungal isolates which was typical of *Trichoderma* species was excised and transferred to 2.5 % PDA amended with 10 mg l^−1^ rifampicin.

Fungal isolates with typical growth of *Trichoderma* species were confirmed to the species level using sequences of the internal transcribed spacer region of the ribosomal RNA gene (ITS rDNA). Mycelia were collected from 7–10 day old cultures of *Trichoderma* species. Mycelia were collected in Eppendorf tubes and kept at −80 °C overnight. This was followed by freeze drying of the collected mycelia.

DNA was extracted from 80 mg freeze dried mycelia following a modified protocol of Lee and Taylor [25]. The ITS rRNA gene region of the fungal isolates was amplified using the universal primers ITS1 and ITS4 [26]. The polymerase chain reaction mixture was carried out using PuReTaq™ Ready-To-Go PCR™ beads (HVD Life Sciences, Vienna, Austria), 0.4 μM ITS1, 0.4 μM ITS4, 25 ng DNA and made up to 25 μl with sterilized distilled water. Thermocycling was run with the following settings: heating at 95 °C (10 min); then 35 cycles of 95 °C (30 s), 55 °C (30 s) and 72 °C (90 s). The final extension was done at 72 °C for 10 min. Amplification of the ITS region was checked out by running 5 μl of the sample on 1.5 % agarose gel in 0.5× Tris-borate-EDTA buffer (TBE) at 120 V for 50 min.

PCR for EF1 gene was conducted using primers EF1-728 F and EF1-986R [20]. The PCR mixture was as described for ITS rDNA. PCR conditions consisted of denaturation at 94 °C for 4 min, followed by 35 cycles of denaturation at 94 °C for 30 s, annealing at 60 °C for 30 s and extension at 72 °C for 60 s. This was followed by extension at 72 °C for 10 min. PCR products were checked by running 5 μl of a PCR product on 1.5 % agarose gel in 5× TBE at 120 V for 40 min.

PCR products were sequenced at Macrogen Inc. (Korea, Seoul) in both senses using the ITS1, ITS4, EF1-728 F and EF1-986R. The resulting forward and reverse ITS and EF1 sequences were aligned and edited using ChromasPro v. 1.41 (Technelysium Pty Ltd). Then, the obtained sequences for each isolate were compared to sequences available at the National Centre for Biotechnology Information (NCBI) (http://www.ncbi.nlm.nih.gov) using BLAST search.

Nine reference ITS sequences representing 9 *Trichoderma* species were obtained from NCBI. The nine representative sequences were aligned with the sequences of the 52 isolates obtained in this study using Clustal W [27]. A neighbour joining tree was constructed using the Kimura 2 parameter evolutionary model (Mega 5) [28]. Consensus trees were generated using 1000 replications (55 % bootstrap criteria).

### AFLP Fingerprinting

Amplified Fragment Length Polymorphism (AFLP) was used to assess genetic diversity within populations of *Trichoderma* species. AFLP fingerprinting was done as described by Al-Sadi et al. [16] using FAM-6-labelled *Eco*RI-AXX selective primers. The primer combinations used in this study were *Eco*RI-AGA/*Mse*I-CAT, *Eco*RI-AGT/*Mse*I-CAT, and *Eco*RI-AGT/*Mse*I-CAA.

DNA restriction and ligation were performed as described by Al-Sadi et al. [16]. The pre-selective amplification reaction mixtures consisted of 0.65 μl of 10 μM each of *Eco*RI + A (5′-GACTGCGTACCAATTCA-'3) and *Mse*I-C (5′-GATGAGTCCTGAGTAAC-′3) primers, 3.7 μl of diluted restriction/ligation mix, PuReTaq^TM^ Ready-To-Go^TM^ PCR beads and Milli-Q water made up to a volume of 25 μl. The cycling profile was conducted as detailed by Al-Sadi et al. [15].

The pre-selective amplification product was diluted by adding 210 μl of TE_0.1_ buffer (20 mM Tris–HCl, 0.1 mM EDTA, pH 8) to the remaining amount. The selective amplification reaction was as above except using 0.13 μl of 10 μM FAM-6 labeled *Eco*RI selective primer, 0.63 μl of 10 μM *Mse*I selective primer, 6 μl of diluted pre-selective amplification and Milli-Q water to a volume of 25 μl. The cycling parameters for the selective amplification were as described by Al-Sadi et al*.* [16]. Fragment analysis of the PCR products from the selective amplification reactions was carried out at Macrogen Inc. (Seoul) using ABI 3730XL (Applied Biosystems, Carlsbad, CA).

### Analysis of AFLP data

Analysis of AFLP data was done to estimate gene diversity, genotypic diversity, genetic distance and genetic differentiation within and among different populations. AFLP alleles in the range of 50 to 500 base pairs (bp) were evaluated using Gene Mapper 4.0 with 0 for absence and 1 for presence of each amplified fragment. The binary data were analyzed using POPGENE version 1.32 [29] which was used to calculate the number of polymorphic loci, Nei’s gene diversity [30] and genetic distance and identity within and among the different populations of *Trichoderma* species*.* Genetic distance based on Nei’s [31] unbiased measurement of genetic distance was also determined between samples and populations of *Trichoderma* species using POPGENE. A dendrogram was constructed based on Nei’s unbiased measures of genetic distance using UPGMA (unweighted pair group method with arithmetic mean; NTSYSpc v. 2.21 m).

Analysis of molecular variance (AMOVA) was used to determine genetic differentiation among the different populations using Arlequin v. 3.1 [32]. AMOVA was conducted only for populations of *T. asperellum* and *T. harzianum* because each species included at least 2 isolates from each substratum.
